# Potential Therapeutic Effects of PPAR Ligands in Glioblastoma

**DOI:** 10.3390/cells11040621

**Published:** 2022-02-10

**Authors:** Rossella Basilotta, Marika Lanza, Giovanna Casili, Giulia Chisari, Stefania Munao, Lorenzo Colarossi, Laura Cucinotta, Michela Campolo, Emanuela Esposito, Irene Paterniti

**Affiliations:** 1 Department of Chemical, Biological, Pharmaceutical and Environmental Sciences, University of Messina, Viale Ferdinando Stagno D’Alcontres, 98166 Messina, Italy; robasilotta@unime.it (R.B.); marika.lanza@unime.it (M.L.); gcasili@unime.it (G.C.); laura.cucinotta@unime.it (L.C.); campolom@unime.it (M.C.); irene.paterniti@unime.it (I.P.); 2 Istituto Oncologico del Mediterraneo, Via Penninazzo 7, 95029 Viagrande, Italy; giulia.chisari@grupposamed.com (G.C.); stefania.munao@grupposamed.com (S.M.); lorenzo.colarossi@grupposamed.com (L.C.)

**Keywords:** glioblastoma, cancer, PPARs, brain, neuro-oncology

## Abstract

Glioblastoma (GB), also known as grade IV astrocytoma, represents the most aggressive form of brain tumor, characterized by extraordinary heterogeneity and high invasiveness and mortality. Thus, a great deal of interest is currently being directed to investigate a new therapeutic strategy and in recent years, the research has focused its attention on the evaluation of the anticancer effects of some drugs already in use for other diseases. This is the case of peroxisome proliferator-activated receptors (PPARs) ligands, which over the years have been revealed to possess anticancer properties. PPARs belong to the nuclear receptor superfamily and are divided into three main subtypes: PPAR-α, PPAR-β/δ, and PPAR-γ. These receptors, once activated by specific natural or synthetic ligands, translocate to the nucleus and dimerize with the retinoid X receptors (RXR), starting the signal transduction of numerous genes involved in many physiological processes. PPARs receptors are activated by specific ligands and participate principally in the preservation of homeostasis and in lipid and glucose metabolism. In fact, synthetic PPAR-α agonists, such as fibrates, are drugs currently in use for the clinical treatment of hypertriglyceridemia, while PPAR-γ agonists, including thiazolidinediones (TZDs), are known as insulin-sensitizing drugs. In this review, we will analyze the role of PPARs receptors in the progression of tumorigenesis and the action of PPARs agonists in promoting, or not, the induction of cell death in GB cells, highlighting the conflicting opinions present in the literature.

## 1. Introduction

Glioblastoma (GB) is the most common and aggressive subtype of malignant brain tumors. This tumor belongs to the large family of gliomas and is also known as grade IV astrocytoma [1]. GB originates from glial cells and astrocytes, which play supporting roles within the central nervous system (CNS) and is characterized by abnormal angiogenesis, apoptosis alteration, and invasiveness. This tumor manifests itself with nonspecific signs and symptoms, which vary according to the size and location of the tumor. Patients often present symptoms of increased intracranial pressure, including focal or progressive headache and neurologic deficits, personality changes, and seizures [2]. Among the genetic risk factors, a set of single nucleotide genetic polymorphisms (SNPs) have been identified, located on different genes (NF1, NF2, IDH1/IDH2, TERT, EGFR, CCDC26, CDKN2B, PHLDB1, TP53, RTEL1), which seem to contribute to gliomagenesis and to the development of all grades and histologies of gliomas [3,4]. The glioblastomas are divided into two broad categories: primary and secondary [5]. Primary GB accounts for 90% of total cases, is more frequent in the elderly population, and has a worse prognosis than its secondary counterpart. Primary GB onco-markers include overexpression of the epidermal growth factor receptor (EGFR) and mutations in the tumor suppressor gene of phosphatase and tensin homolog (PTEN) and the telomerase reverse transcriptase promoter (TERT) [6]. Secondary GB constitutes 5% of cases and develops from astrocytomas with a lower degree of malignancy, affects younger patients and is related to mutations in isocitrate dehydrogenase 1 and 2 (IDH 1 and 2) and tumor protein 53 (P53) [7]. It is a tumor characterized by an extraordinary intra-tumoral heterogeneity which often results in the inability of traditional therapies to obtain long-term remissions. Glioblastomas, in fact, differ in phenotypic properties, including transient quiescence, self-renewal, adaptation to hypoxia, and resistance to therapy-induced DNA damage. For this reason, the development of new personalized treatment strategies for GB represents both a preclinical and clinical challenge [8]. To date, the causes and physiopathology of GB are unknown. Current therapy of choice consists of surgical resection or biopsy, followed by radiotherapy and concomitant chemotherapy with temozolamide (TMZ). TMZ, the gold standard anticancer drug for the treatment of GB, belongs to the class of orally administered alkylating agents and improves prognosis by increasing median patient survival. The approved standard therapy consists of a daily dose of 150 to 200 mg per square meter of body surface area for 5 days of each 28-day cycle. Daily therapy at a dose of 75 mg per square meter for up to seven weeks is safe; at these doses, TMZ depletes the DNA repair enzyme MGMT, resulting in tumor tissue shrinkage, an effect associated with longer survival among glioblastoma patients [9,10]. Considering the aggressiveness of GB and the low efficacy of therapeutic strategies, it is currently necessary to identify new therapeutic targets able to reduce or arrest the progression of GB. GB aggressiveness appears to be related to the presence of tumor stem cell populations called GSCs that contribute to GB malignancy by promoting tumor growth, angiogenesis, and therapeutic resistance. Unlike well-differentiated tumor cells, which show limited replicative potential, GSCs can proliferate indefinitely and spread to tissues and organs distant from the primary tumor site, becoming responsible for initiation, growth of metastases and resistance to therapy. According to some studies, the preferential overexpression of nicotinamide N-methyltransferase (NNMT) in GSC, a cytosolic enzyme involved in the biotransformation of many xenobiotics, causes the exhaustion of the methyl donor S-adenosyl methionine (SAM), with consequent hypomethylation of the GB DNA, causing the translation of the tumor towards a mesenchymal phenotype and accelerating its growth [11,12]. The main mechanism of resistance to TMZ therapy is related to the overexpression of O-6-methylguanine-DNA methyltransferase (MGMT), a gene located on chromosome 10q26 that codes for a DNA repair protein, which removes alkyl groups from the O-6 position of guanine, an important alkylation site. High levels of MGMT in tumor cells, therefore, induce the formation of a resistant phenotype by reducing the efficacy of alkylating agents such as TMZ, thus leading to therapeutic failure. MGMT in its methylated and therefore inactive form represents a molecular marker of clinical relevance, associated with the response to alkylating chemotherapy and survival of patients with GB [13,14,15].

Peroxisome proliferator-activated receptors (PPARs) are ligand-inducible transcription factors that belong to the superfamily of proteins called nuclear hormone receptors (Nrs), to which steroid, thyroid, and retinoid receptors also belong. Three isoforms of PPAR have been identified: PPAR-α, PPAR-β/δ, and PPAR-γ, differently expressed based on the physiological role, tissue distribution, and specificity of the ligands. Each of the isoforms activates or suppresses different genes involved primarily in the metabolism and homeostasis of fats and carbohydrates, as well as in proliferation and cell differentiation, inflammation, and cancer [16]. 

All three isotypes of PPAR are co-expressed in the central nervous system (CNS), but their function in this tissue is still poorly understood. Some studies show that, at the level of CNS, PPARs are involved in lipid metabolism, neuronal differentiation, and death, as well as inflammation and neurodegeneration. Observations both in vitro and in vivo show that PPAR-β/δ is the prevalent isoform in neurons of different brain areas, while PPAR-α is expressed at very low levels, predominantly in astrocytes, and appears to be involved in the neurotransmission of excitatory amino acids [17]. The expression of PPAR-γ in the brain has been extensively studied in relation to inflammation and neurodegeneration [18]. 

Although PPAR receptors are known for their role in lipid metabolism and glucose homeostasis, a lot of research in the literature has demonstrated the contribution of these receptors in tumors and GB biology [19,20]. 

Preclinical and clinical studies have shown beneficial effects of PPAR agonists against GB growth, inhibiting the invasion and motility of glioma cells and thus increasing the chance of survival [21]. The purpose of this review is to summarize the role of PPARs in GB, focusing on the antitumor action of their synthetic and natural ligands, in order to consider them as potential additional treatments to conventional therapies. 

## 2. Peroxisome Proliferator-Activated Receptors

Peroxisome proliferator-activated receptors (PPARs) are ligand-activated transcription factors involved in various processes at the cellular level and in the regulation of lipid, carbohydrate, and amino acid metabolism. PPARs belong to the superfamily of nuclear hormone receptors (Nrs), which after interacting with specific ligands (synthetic or non-synthetic), translocate to the nucleus where they modify their conformation and regulate gene transcription through the differential recruitment of cofactors and enzymes modifying the histone [22]. Once translocated to the nucleus, PPARs interacts with retinoid X receptors (RXR), peroxisome proliferator-activated receptor gamma-coactivators (PGC), steroid receptor coactivators (SRC), and CREB binding protein (CBP/p300), then bind to the sequences of the peroxisome proliferating receptor element (PPRE) and consequently initiate the transcription of target genes involved in various physiological processes [23]. PPARs, in fact, control gene expression involved in energy homeostasis, lipid metabolism, and adipogenesis: they represent the main receptors for dietary fats such as oleic and linolenic acids and for many lipid metabolites, for example, prostaglandin J2, 8S-hydroxyheicosatetraenoic acid (8-HETE), and oxidized phospholipids. The altered expression of PPARs is also related to the onset of many diseases such as type 2 diabetes, dyslipidemia, obesity, atherosclerosis, and metabolic syndrome [21]. PPARs are also expressed in the cardiovascular system (endothelial cells, vascular smooth muscle cells, monocytes, and macrophages), and many clinical and preclinical studies have shown the significant role of PPARs also in cardiovascular diseases [24]. Today, increasing attention has been paid to the critical role of PPARs in inflammation and cancer; numerous studies have, in fact, highlighted the overexpression of PPARs in many human solid tumors [25]. 

**Figure 1:**
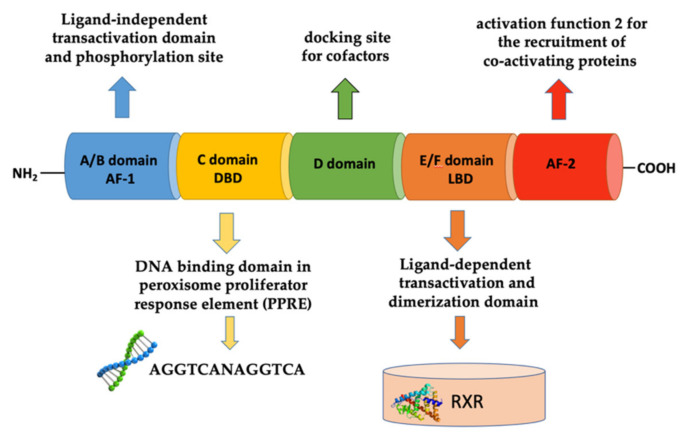
Schematic representation of the structure of PPAR receptor.

Studies based on X-ray crystallography and molecular modeling have shown that the structure of PPARs consists of six functional domains, from A to F [26]. The N-terminal portion of PPARs exhibits the ligand-independent transactivation domain (or A/B domain), also called activation function 1 (AF-1) responsible for transcriptional activation, followed by the C domain, also called DNA binding domain (DBD), involved in recognition of the DNA sequence in the promoter region of genes known as peroxisome proliferator response element (PPRE). The C-terminal of the PPAR receptor, on the other hand, contains the D domain, which thanks to its flexibility acts as a docking site for the cofactors and the E/F (or LBD) domain, which is responsible for the specificity of the ligand and for dimerization of the receptor with the retinoid X receptors (RXR). The dimerization domain is critical for the formation of heterodimers with the retinoic acid receptor α (RXRα), an important prerequisite for PPARs to bind DNA in regions containing the DNA sequence AGGTCANAGGTCA. The C-terminal also possesses the AF-2 activation domain, that after binding with the ligand, synergizes with AF-1 and undergoes conformational modifications allowing the recruitment of co-activating proteins p300, CREB binding protein (CBP), or coactivator steroid receptor 1 (SRC1), important for the transcriptional activation of their target genes (Figure 1) [27]. 

### PPARs Isoforms: Tissue Distribution and Biological Activity 

The PPAR family includes three different subtypes which differ in terms of tissue distribution, affinity for ligands, and biological activity. They are called PPAR-α, PPAR-β/δ, and PPAR-γ. All isoforms participate differently in lipid homeostasis and glucose regulation (energy balance), but each of them is capable of suppressing or activating different genes [28].

**Figure 2:**
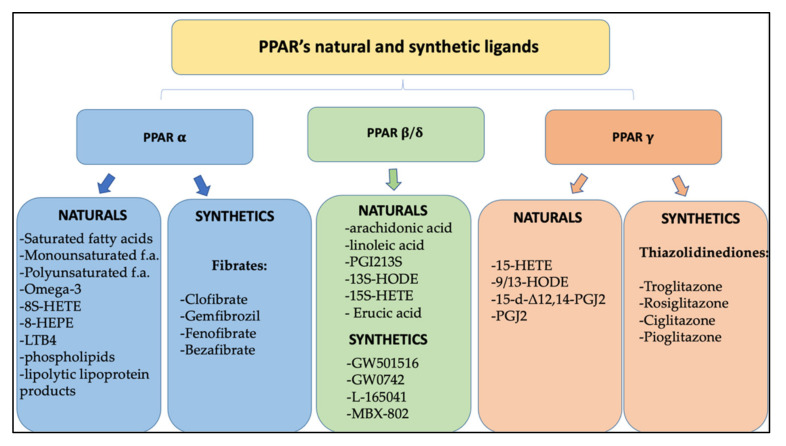
Classification of natural and synthetic ligands of PPARs receptors.

Based on their tissue distribution, PPAR-α receptors are mainly expressed in brown adipose tissues, skeletal muscle, kidney, heart, liver, and intestinal mucosa and are involved in glucose metabolism and homeostasis and in the oxidation of fatty acids [29]. The PPAR-α receptor is activated by natural ligands, including saturated, monounsaturated, and polyunsaturated fatty acids and their metabolites such as 8S-HETE and 8-HEPE, leukotrienes B4 (LTB4), oxidized phospholipids, and lipolytic lipoprotein products. Among these omega-3 fatty acids, being highly polyunsaturated, oxidize easily and stimulate PPAR-α, causing a decrease in lipid levels and the elimination of triglycerides from the plasma with a consequent increase in the levels of high-density lipoprotein cholesterol (HDL) and reduction of inflammation and arteriosclerosis in the cardiovascular system. Furthermore, an important anti-inflammatory effect also derives from the inhibition of the oxidation of omega-3 fatty acids, mediated by NF-κB in a PPAR-α-dependent pathway [30]. Synthetic PPAR-α ligands are represented by fibrates (clofibrate, gemfibrozil, fenofibrate, and bezafibrate), a class of lipid-lowering drugs that are used in the treatment of hypertriglyceridemia. Through the activation of PPAR-α, they cause an increase in gene expression involved in the β-oxidation of fatty acids leading to the reduction of triglyceride-rich lipoproteins in the serum and to the increase of HDL cholesterol, slowing the progression of arteriosclerosis and reducing cardiovascular events (Figure 2) [31].

The PPAR-β/δ receptor consists of 441 amino acids, with a molecular weight of 49.9 kDa and is ubiquitously expressed in almost all tissues, including the liver, intestines, kidneys, abdominal fat, skeletal muscle, brain, and pancreas. Like the other members of the PPAR family, it mainly intervenes in the metabolism of lipids, participating in the oxidation of fatty acids, both at the level of adipose tissue, reducing adiposity, and consequently preventing the development of obesity, both at the level of skeletal muscles and heart and regulating the concentrations of cholesterol and blood glucose [32]. Moreover, many studies have revealed a large expression of PPAR-β/δ in the central nervous system (CNS), in particular at the level of neurons, astrocytes, oligodendrocytes and in microglia cells, suggesting the role of these receptors as targets for neuroinflammation and neurodegeneration [33]. Although PPAR-β/δ has a smaller binding domain (LBD) than other members of the PPAR family, it has the ability to bind many endogenous ligands, but with relatively low selectivity. Natural ligands include polyunsaturated fatty acids (arachidonic and linoleic acids) and their metabolites like prostacyclin PGI2, 13S-hydroxyoctadecadienoic acid (13S-HODE), and 15S-hydroxyheicosatetraenoic acid (15S-HETE), which could have promising applications in cardiomyopathy diabetic. Synthetic agonists (GW501516, GW0742, L-165041, and MBX-802) that have been developed and proposed as treatments for obesity and metabolic syndrome have not been used for clinical trials due to their carcinogenic effects, so none of them have been approved for clinical use to date (Figure 2) [34]. Despite controversial data on its pro-tumorigenic versus anti-tumorigenic action, few results suggest that PPAR-β/δ activation can reduce the growth of neuroectodermal tumors, including glioblastomas [35]. 

**Figure 3:**
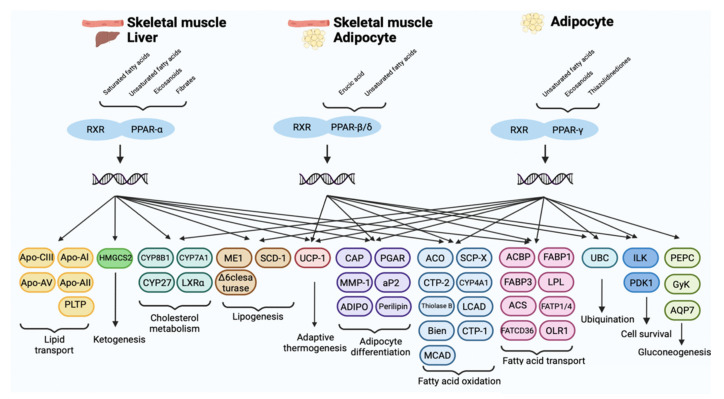
A schematic representation at cellular level of PPARs signaling.

The PPAR-γ receptor is expressed in many human tissues and has three different isoforms: PPAR-γ1, γ2, and γ3. PPAR-γ1 is ubiquitously expressed in all human cells, PPAR-γ2 has been predominantly detected in white and brown adipose tissue, as well as in the large intestine and spleen, while PPAR-γ3 expression is limited. PPAR-γ is poorly expressed in the central nervous system (CNS), but it was found in different cell types such as neurons, astrocytes, oligodendrocytes, and microglia. Its physiological role includes the regulation of adipogenesis and the levels of adipokines such as adiponectin, TNFα, MCP-1, and resistin, and it is also involved in the energy balance and lipid biosynthesis. Thanks to the size of its binding cavity, the PPAR-γ receptor is able to bind a large variety of natural or synthetic lipophilic acids. The natural modulators of PPAR-γ are mainly unsaturated fatty acids and their metabolites, including 15- hydroxyeicosatetraenoic acid (15-HETE), 9- and 13-hydroxyoctadecadienoic acid (9/13-HODE), 15-deoxy- Δ12,14-prostaglandin J2 (15-d-Δ12,14-PGJ2), and prostaglandin PGJ2, whose physiological role is still to be clarified [36]. Synthetic PPAR-γ ligands belong to the thiazolidinediones class (TZD), including troglitazone, rosiglitazone, ciglitazone, and pioglitazone, and they are known as insulin-sensitizing drugs. By activating the PPAR-γ receptor, in fact, they reduce the hepatic production of glucose and prolong the function of pancreatic cells, preventing apoptosis of β cells (Figure 2). TZD are used in the treatment of type 2 diabetes, as they increase insulin sensitivity and improve glucose control, but their use is limited by important adverse events, including the risk of bone fracture and congestive heart failure [37] (Figure 3). 

## 3. Role of PPARs in Tumors

Certain PPAR-related metabolic alterations, such as obesity and type 2 diabetes, have been identified as risk factors for cancer cell proliferation and thus tumor progression. Hence, research is currently focused on using PPARs as targets for cancer therapy, and a few studies have focused on understanding their role in human cancer and in the antitumor activity of their natural and synthetic agonists. Furthermore, current studies have revealed conflicting results on the role of the different isoforms of PPARs in various tumor types; most investigations have shown that PPAR-β/δ activation is linked to tumor progression, while PPAR-α and PPAR-γ are associated with an antitumor action [38]. Some in vitro studies on breast cancer cells SUM149PT and SUM1315MO2 have shown interesting results in the context of nuclear PPAR-α receptor signaling, demonstrating that its activation by clofibrate agonist suppresses the inflammatory activity of cyclooxygenase-2 (COX-2) and 5-lipoxygenase (5-LO) and determines the decrease in the secretion of prostaglandin-E2 (PGE2) and leukotrienes-B4 (LB4), effectively inhibiting cell survival and cell cycle-related kinases [39]. Another study conducted in vivo showed that the combination of clofibric acid (PPAR-α agonist) and pioglitazone (PPAR-γ agonist) reduces angiogenesis, induces apoptosis, significantly decreasing the expression of COX-2 and VEGF, through inhibition of activator protein-1 (AP-1) and suppressing the growth of solid ovarian tumors [40]. Evidence suggests that certain PPAR-α ligands, including bezafibrate and fenofibrate, may act as potential chemopreventive agents in colon carcinogenesis by reducing intestinal polyp formation in Apc-deficient mice, by inhibiting AOM/DSS-induced colon carcinogenesis. However, the exact mechanism by which PPAR-α activation suppresses colon carcinogenesis is still unclear [40]. Moreover, only a few studies support the pro-carcinogenic role of PPAR-α. For example, some investigations carried out on human breast cancer cell lines (MDA-MB-231) have shown that inhibition of PPAR-α by the antagonist GW6471 leads to an impairment of the mevalonate pathway and a substantial reduction in cholesterol and lipid droplets. This causes a consequent perturbation of lipid metabolism and cell death, influencing the pathways involved in the control of proliferation, such as the pathways involving the Rho Family and YAP/TAZ and Wnt/β-catenin signaling [41]. The conflicting results presented in the literature regarding the action of PPAR-α on tumor progression could be due to the different evaluation conditions, cell lines used, the stage of differentiation, the cellular context, and the microenvironment. 

The role of PPAR-β/δ on cell proliferation, induction of angiogenesis, and cell death has been extensively investigated. Although there are conflicting opinions on the effects of PPAR-β/δ activation for cancer progression, most reports suggest that its stimulation could have pro-tumorigenic effects [42]. PPAR-β/δ exerts proangiogenic effects, directly or indirectly modulating downstream proinflammatory or proangiogenic molecules that act on multiple cell types in the tumor microenvironment, promoting cancer progression and metastasis [43]. The increased expression of PPAR-β/δ mRNA in colon cancers has been attributed to APC-β-catenin-TCF4-mediated transcription, similar to the well-known target gene β-catenin-TCF4 CCND1, which encodes cyclin D1. This supports the hypothesis that PPAR-β/δ regulates genes that increase cell proliferation and promote colon carcinogenesis [44]. Consistent with these results, PPAR-β/δ has been shown to strongly potentiate aberrant activation of β-catenin in mouse genetic models of human CRC, with representative APC mutations and overexpression or deletion of PPAR-β/δ in intestinal epithelial cells (IEC), activating pro-invasive pathways to promote CRC tumorigenesis. These results have demonstrated that PPAR-β/δ strongly accelerates APC mutation-driven CRC progression and invasion through multiple important pro-tumorigenic pathways, including BMP7/TAK1/-catenin, PDGFRβ, AKT1, EIF4G1, and CDK1 [45]. The role of PPAR-β/δ in enhancing cell proliferation was supported by a further study performed on human liposarcoma cells (SW872, T778), in which an increase in cell proliferation was observed in response to PPAR-β/δ activation by the agonist GW0742, which appears to be caused by leptin repression, suggesting the potential therapeutic use of PPAR-β/δ antagonists for the treatment of unresectable liposarcomas [46]. PPAR-β/δ seems to be overexpressed in breast cancer cells and the elevated levels appear to correlate with greater migratory and metastatic properties. PPAR-β/δ mediates these effects by mechanisms including increased expression of antioxidant proteins such as catalase and increased AKT-mediated survival signaling after prolonged nutrient deprivation [47]. 

Among the three isoforms, PPAR-γ is certainly the most studied in tumors. It is, in fact, expressed in a wide variety of cancers and its role in cancer initiation/progression has long been debated. The literature suggests that PPAR-γ plays a key role in tumorigenesis as a tumor suppressor. Indeed, PPAR-γ activation by many agonists has shown antiproliferative and proapoptotic actions in colon, esophageal, thyroid, breast, lung, and prostate cancers [48,49,50]. The mechanism by which it induces tumor cell growth arrest appears to be related to the PPAR-γ-dependent upregulation of the tumor suppressor gene and of the homologous tensin phosphatase (PTEN), which inhibits the phosphorylation of PI3-kinase and AKT by reducing cell migration and proliferation. To the antiproliferative effects is also added the downregulation of the anti-apoptotic protein B-cells/lymphoma 2 (Bcl-2), the anti-angiogenic activity through the inhibition of VEGF and its receptors in various cells and anti-inflammatory properties through NFκB-mediated inhibition of gene transcription. Furthermore, PPAR-γ appears to hinder the formation of metastases through the inhibition of the epithelial–mesenchymal transition (EMT), a process by which epithelial cells lose their cell polarity and cell–cell adhesion, and gain migratory and invasive properties. Consistent with these findings, growing evidence suggests that PPAR-γ overexpression has important suppressive activities in colorectal cancer growth. In fact, preclinical studies currently analyze new PPAR-γ agonists, capable of inhibiting the Wnt/β-catenin pathway, acting as modulators of PPAR-γ signaling, and interfering with related pathways in order to provide new therapies for CRC [51]. PPAR-γ thiazolidinediones (TZD) ligands have been shown to counteract the stimulatory effects of leptin on breast cancer growth in vivo and in vitro models. The results show that PPAR-γ activation inhibited cell proliferation and prevented the development of leptin-induced MCF-7 tumor xenografts [52]. 

PPAR-γ expression was studied in patients with esophageal squamous cell carcinoma (ESCC), on which the antiproliferative effect and mechanism of action of the PPAR-γ agonist, ephatutazone, were investigated. Ephatutazone has been shown to cause a 49.6% reduction in the proliferation of xenotransplanted ESCC cells through a mechanism that involves the regulation of p21Cip1 protein levels in the nucleus by inactivating the Akt signal and dephosphorylating p21 to Thr145, without altering the transcriptional activity of p21Cip1 [53]. Another study found a significant increase in PPAR-γ expression in NSCLC cell lines by both immunohistochemistry and Western blotting. In addition, significant antitumor activity was observed both in vivo and in vitro in response to treatment with troglitazone or pioglitazone, with a correlated reduction in metastases [54]. 

Although many results strongly support the role of PPAR-γ as a tumor suppressor, other studies, on the contrary, argue that it plays a role as a tumor promoter. Indeed, a recent animal experiment of prostate orthotransplantation identified the involvement of PPAR-γ in the upregulation of the AKT3-PGC1α axis. PPAR-γ seems to increase the regulation of AKT3, destabilizing CRM1 and favoring the localization of PGC1α in the nucleus with a consequent increase in mitochondrial function and ATP levels. The high levels of ATP appear to be related to promoting tumor growth and metastasis [55]. Further evidence reveals that PPAR-γ activation reduces TXNIP expression in human melanoma cells (A375 and C8161), affecting the expression of proteins of particular relevance to melanoma cell invasiveness, such as integrin alpha-v/beta-3 and TIMP-2, resulting in melanoma progression to a metastatic phenotype [56].

### 3.1. Role of PPAR-α Agonists in GB

**Figure 4:**
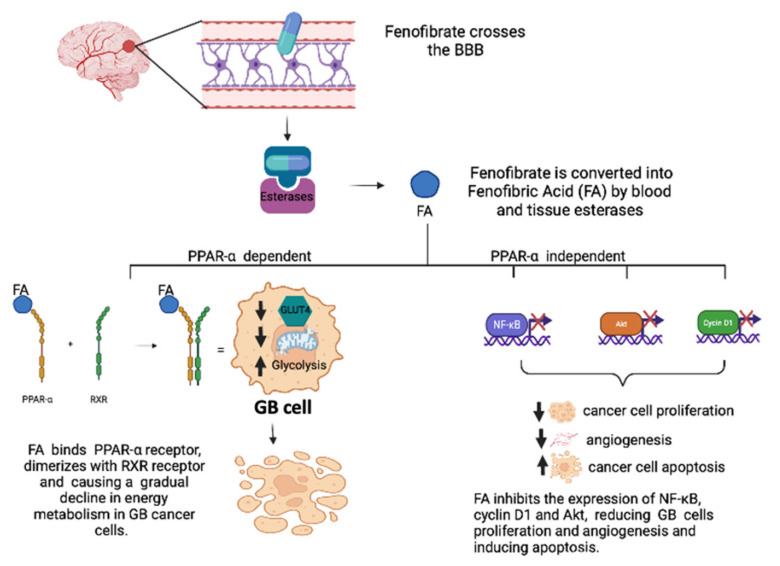
An exemplified overview of fenofibrate’s PPAR-dependent and PPAR-independent mechanisms of action in GB tumoral cells.

Conflicting results emerged regarding the role of PPAR-α in GB tumorigenesis. Studies have examined the expression of PPAR-α protein and PPAR-α mRNA in primary wild-type human IDH1 GB, arguing that their overexpression in GB is related to the degree of glioma malignancy [57]. This overexpression appears to be accompanied by the significant increase in 30-hydroxy-30-methylglutaryl-CoA reductase (HMGCR), the cholesterol biosynthesis-limiting enzyme (CHO), that catalyzes the formation of mevalonate (MVA). These results indicate that PPAR-α, by regulating CHO metabolism, is involved in the strong alteration of lipid homeostasis observed in gliomas and could therefore drive the tumorigenesis process. In fact, the use of a compound derived from N-phenylsulfonyl (AA452), capable of blocking the activation of PPAR-α, has determined a strong effect on cell viability, reducing cell proliferation and migration and therefore decreasing tumor invasiveness [58]. Other evidence discusses that the expression of PPAR-α receptors is negatively correlated to the degree of malignancy of the glioma, in fact, its activation suppresses the proliferation of tumor cells, delays the cell cycle to the G1 phase and induces apoptosis and the accumulation of species reactive oxygen (ROS) in U87 cells [59,60]. The anticancer effects of the PPAR-α agonist fenofibrate have been demonstrated in several cell lines of colon, breast, endometrial, skin, medulloblastoma, and melanoma cancers [61,62]. Among the PPAR-α ligands tested in GB, fenofibrate received the most attention, due to its capacity to cross the blood brain barrier (BBB) and has an established anti-inflammatory activity and limited toxicity and a better side effect profile [63]. In order to activate the PPAR-α receptor, fenofibrate must first be converted into fenofibric acid (FA) by blood and tissue esterases. Once converted FA then binds to the PPAR-α receptor and triggers the expression of numerous metabolic enzymes involved in the oxidation of fatty acids and reduces glucose uptake by repressing the insulin-dependent glucose transporter GLUT4. This metabolic switch could explain the mechanism by which fenofibrate initiates a gradual decline in energy metabolism in cancer cells. However, the anticancer effects of fenofibrate are more pronounced than other PPAR agonists and may be due to its accumulation in the mitochondrial fraction of human GB cells, which respond with a sudden and severe inhibition of mitochondrial respiration and an immediate increase but transient glycolysis, effectively triggering an energy catastrophe in GB cells with significantly reduced toxicity in normal astrocytes [64]. The anticancer effects of fenofibrate are therefore very complex and cannot be explained simply by activation of PPAR-dependent transcription, but it is, therefore, necessary to consider PPAR-independent mechanisms. Some investigations have also confirmed that fenofibrate is a neuroprotective agent; the results obtained in vitro on high-grade glioma (HGG) cell lines U87 and U343 (p53 wild-type), U251 and T98 (p53 mutant), confirmed the antiproliferative and pro-apoptotic effects of fenofibrate demonstrating inhibition of NF-κB expression, cyclin D1, and Akt (Figure 4). Akt is well established as a therapeutic target for HGG, and there are a number of Akt inhibitors being evaluated as an adjunct treatment for HGG [65]. The transcription factor Fork-head box O1 (FoxO1) is an Akt substrate that plays a key role in tumor suppression by promoting transcriptional activation of p27kip. A recent study showed that fenofibrate by activating the PPARα/FoxO1/p27kip pathway could actually induce the death of human U87MG glioma cells, causing cell cycle arrest in the G0/G1 phase [66]. The activation of the PPAR-α receptor by the agonist fenofibrate attenuates the signaling responses of the IGF-IR, a factor that helps to support malignant growth and invasion of glioma cells and causes the accumulation of reactive species of oxygen (ROS), loss of mitochondrial membrane potential and a deficit in ATP production, which together may explain the severe impairment of glioma cell motility [67]. A recent study demonstrated that fenofibrate modulates the expression of hypoxia-inducible factor-1 alpha (HIF-1α), an overexpressed transcription factor under hypoxic conditions in human GB samples. HIF-1α is involved in the transactivation of genes involved in the altered metabolism, causing the accumulation of metabolites in the tumor environment, thus contributing to the growth and increase of the aggressiveness of GB. Fenofibrate inhibits the expression of HIF-1α by activation of HO-1 via the AMPK pathway. Furthermore, the activation of HO-1 involves the upregulation of SIRT1, causing its translocation into the nucleus, with consequent deacetylation of HIF-1α and inhibition of transcriptional activity [68].

A dual agonist PPAR-α/PPAR-γ, also called TZD18, inhibited cell growth and induced apoptosis in human GB T98G cells, through the activation of caspase-3 and down-regulation of the expression of Bcl-2, suggesting that TZD18 may have a therapeutic role in the treatment of human GB [69]. 

Moreover, PPAR-α and PPAR-γ agonists have the ability to selectively upregulate catalase expression on human astrocytes. In fact, both C6 GB cells and normal astrocytes were treated with PPAR-α and PPAR-γ agonists, showing a significant increase in catalase expression only in normal astrocytes, while, on the contrary, they failed to increase catalase expression in glioma cells. These results are promising because current data support the concept that selective manipulation of catalase gene expression and/or activity can provide greater protection of astrocytes from H_2_O_2_-induced damage and consequently can improve normal tissue survival during radiotherapy [70].

### 3.2. Role of PPAR-β/δ in GB

Although PPAR-β/δ agonists have been shown to cross the blood–brain barrier and modulate oxidative stress and proinflammatory responses associated with acute and chronic CNS disorders, overall, there are only a few studies evaluating the action of the PPAR-β/δ receptor in brain tumors [71]. The effects of a PPAR-β/δ ligand, namely erucic acid (EA), an omega-9 fatty acid, were investigated. EA has been shown to block the growth of C6 glioma cells and also reduce the cardiotoxicity of doxorubicin, thus suggesting that the combination of systemic EA with DOX-chemotherapy can reduce DOX concentrations in the systemic circulation, hinder toxic interactions and induce selective killing of glioma cells [72]. However, these results are too small to evaluate the role of the receptor PPAR-β/δ in brain tumors, especially considering the prevalence of the results in the literature that instead supports a procarcinogenic action in other types of tumors.

### 3.3. Role of PPAR-γ Agonists in GB 

In addition to well-defined metabolic actions, PPAR-γ agonists exhibit various antineoplastic effects and induce cell death by apoptosis in various brain tumor cell lines. There are several possible mechanisms by which PPAR-γ agonists inhibit cell proliferation, such as induction of cell cycle arrest in the G0/G1 phase, a reduction in MYC levels upstream of the S phase transition, as well as possible down-regulation of CCND1 (cyclin D1) and associated cyclin-dependent kinases, but also the upregulation of cyclin-dependent kinase inhibitors CDKN1A, CDKN1B, and CDKN2B [21]. PPAR-γ agonists also blocking the Janus kinase/signal transducer and transcription activator (JAK/STAT) pathway, inhibit the expansion of CD133 + brain tumor stem cells (BTSC is also called tumor-initiating brain cells). In vivo and in vitro models have shown that inhibition of JAK2 (upstream regulator of STAT3) by the agonist PPAR-γ troglitazone promotes the slowing of the progression of GB disease, causing the phosphorylation of STAT3 tyrosine 705 and leading to the down-regulation of CCND1 and BCL2L1 (B-cell lymphoma extra-large protein 2) [73]. 

Agonists PPAR-γ, PGJ2, and rosiglitazone, have been shown to inhibit the proliferation of GB cell lines (U87-MG) through G2/M arrest and promoting the induction of programmed cell death [74]. These results are consistent with another study, in which evaluated growth inhibition and induction of apoptosis by another TZD, ciglitazone. Ciglitazone in addition to inducing cell cycle arrest, causes a significant reduction in the activity of telomerase, an enzyme that is constitutively active in most tumor cells, in human GB cell lines U-87 MG and U-118 MG [75]. Other data suggest that ciglitazone is able to induce PPAR-γ-independent apoptotic cell death in human T98G glioma cells by down-regulation of Akt and reduction of mitochondrial membrane potential (MMP), an effect that was accompanied by a down-regulation of Bcl-2 expression and an increase in Bid cleavage [76]. A study shows that the PPAR-γ receptor is an important positive regulator of the expression of CIDEA, a member of cell death-inducing DFFA-like effector (CIDE) protein family. In fact, it has been shown that PPAR-γ inhibition improves CIDEA expression, triggering glioma cell apoptosis and a decrease in HIF-1α activation, justifying further investigations aimed at evaluating the efficacy of PPAR-γ inhibitors as an effective anti-glioma therapeutic strategy [77]. Another TZD, Pioglitazone, showed anticancer efficacy on human glioma cells (U87MG, T98G, and U251MG) in vitro. Pioglitazone-induced inhibition of glioma cell proliferation and invasion occurred in a PPAR-γ-dependent manner and is in agreement with its ability to dramatically reduce β-catenin expression and transcriptional activity, resulting in decreased cell proliferation, migration, and apoptosis. These results indicate that PPAR-γ activation induces suppression of glioma cell turnover [78]. The agonist PPAR-γ Pioglitazone has also been shown to increase the functional expression of the glutamate transporter EAAT2 in glioma cells, preventing excitotoxic damage and glutamate-mediated seizures related to glioma [79]. In addition to the antineoplastic and anticonvulsant effects of pioglitazone, there is also the demonstration of its ability to cross the blood–brain barrier after oral and intracerebral administration in a human glioma xenograft model, suggesting its possible use as an additional candidate in the current regimen for double mechanistic efficacy in subtherapeutic doses to avoid associated adverse effects [80]. 

Clinical studies suggest a potential protective effect of the PPAR-γ agonists pioglitazone or rosiglitazone in diabetic patients with GB. The results show that diabetic patients with GB who had been treated with PPAR-γ agonists showed an increase in median survival of 19 months compared to patients who received standard treatment only [81]. In addition, a Phase 1 clinical study conducted on patients with primary and metastatic brain tumors treated with radiotherapy highlighted the protective role of pioglitazone in the prevention of radiation-induced cognitive decline (RICD) and its good tolerability at the 45 mg dose, not showing dose-limiting toxicity (DLT), which may be suggested for efficacy studies [82] (Table 1).

## 4. Conclusions and Future Prospects

In this review, a detailed analysis was carried out in order to summarize the role of the PPAR receptor family in GB, a brain tumor characterized by high aggression. The PPARs family includes three different subtypes PPAR-α, PPAR-β/δ, and PPAR-γ, which differ in terms of tissue distribution, affinity for ligands, and biological activity, and which participate differently in the maintenance of lipid and glucose homeostasis [83]. Their role in the progression and differentiation of cancer cells in different types of solid tumors is also widely studied, even if often the data present in the literature report conflicting opinions. Nevertheless, most investigations have shown that PPAR-β/δ activation is related to tumor progression, whereas PPAR-α and PPAR-γ are associated with antitumor action [84]. Based on these findings, considerable interest has been shown in PPAR ligands as potential therapeutic agents in the treatment of gliomas; however, the molecular mechanisms underlying the suppression of carcinogenesis in gliomas, determined by PPAR activation have not yet been fully elucidated. Particularly among the fibrates, a PPAR-α ligands, fenofibrate has received the most attention due to its capacity to reduce the proliferation of GB cells through both PPAR-dependent and PPAR-independent mechanisms [85]. The thiazolidinediones (TZD) class, PPAR-γ ligands known as insulin-sensitizing drugs, including troglitazone, ciglitazone, rosiglitazone, and pioglitazone have also been shown to interact with several pathways involved in the induction of cell death in GB cells [86]. On the other hand, little attention has been paid to the PPAR-β/δ ligands, probably due to the conflicting evidence in the literature regarding its pro-carcinogen action, so further studies would be needed to clarify its function in this context. Finally, there are clinical studies aimed at evaluating the efficacy and safety of these ligands in patients with GB, but further results are certainly needed in order to be able to suggest PPAR ligands as potential treatments in the therapy of GB. 

## Figures and Tables

**Table 1:** This table summarizes the studies that analyze the effects of PPAR ligands.

Drug	Target	Effects	References
Fenofibrate	PPAR-α	-Repression of GLUT4 -Inhibition of NF-κB, cyclin D1 and Akt expression	[63,64,65,66,67,68]
TZD18	PPAR-α/PPAR-γ	-Activation of caspase-3 -Reduction of Bcl-2 expression	[69]
PGJ2 Rosiglitazone	PPAR-γ	-Induction of G2/M arrest	[74]
Ciglitazone	PPAR-γ	-Reduction of telomerase activity -Reduction Akt and Bcl-2 expression	[75,76]
Pioglitazone	PPAR-γ	-Reduction β-catenin expression -Increase EAAT2 expression	[78,79,80]

## Data Availability

Not applicable.
